# Wheat Bread Supplemented with *Agaricus bisporus* Powder: Effect on Bioactive Substances Content and Technological Quality

**DOI:** 10.3390/foods11233786

**Published:** 2022-11-24

**Authors:** Aneta Sławińska, Bartosz G. Sołowiej, Wojciech Radzki, Emilia Fornal

**Affiliations:** 1 Department of Plant Food Technology and Gastronomy, Faculty of Food Sciences and Biotechnology, University of Life Sciences in Lublin, Skromna 8, 20-704 Lublin, Poland; 2 Department of Dairy Technology and Functional Foods, Faculty of Food Sciences and Biotechnology, University of Life Sciences in Lublin, Skromna 8, 20-704 Lublin, Poland; 3 Department of Bioanalytics, Medical University of Lublin, Jaczewskiego 8b, 20-090 Lublin, Poland

**Keywords:** functional breads, mushroom powder, vitamin D_2_, ergocalciferol, polyphenols, antioxidants, colour, texture

## Abstract

Supplementation of food products with mushroom powder increases their health-promoting value, but at the same time affects technological quality, which often play a key role for consumers. The aim of the research was to determine the effect of adding freeze-dried white and brown button mushrooms (2.5% and 5%) to wheat bread on its health-promoting properties such as antioxidant activity (DPPH, FRAP), total polyphenols and vitamin D_2_ content and as well as the technological quality as colour and texture. The breads were supplemented with mushroom lyophilisates, which were exposed to UVB radiation in order to increase their vitamin D_2_ content. The content of total polyphenols and antioxidant properties were determined spectrophotometrically, and the content of vitamin D_2_ by ultra-high performance liquid chromatography coupled to triple quadrupole spectrometer (UHPLC/MS/MS analysis). Colour parameters were determined in the CIE-Lab system and texture profile analysis (TPA) and sensory evaluation of the baked products were performed. The addition of dried mushrooms significantly increased the content of bioactive compounds (total polyphenols, vitamin D_2_) and the antioxidant properties of bread. A small addition of mushrooms caused a significant change in the basic technological quality of breads (colour parameters, specific volume, hardness, cohesiveness, springiness). At the same time, supplementation with mushroom lyophilisates has a positive effect on most analysed attributes in the nine-point hedonic scale. Based on the conducted research, it can be concluded that mushroom lyophilisates can be a valuable raw material for the fortification of bread, which is a good matrix and carrier of substances with documented biological activities.

## 1. Introduction

Mushrooms are valued not only for their taste and aroma, but also for the presence of many bioactive substances. These compounds include: vitamins from the B-group [1] and vitamin D_2_ [2], minerals [3], phenolic compounds [4], terpens [5], sterols [6] or β-glucans [7]. For this reason, mushrooms are considered as functional food and are more and more often used to increase the nutritional or health-promoting value of various food matrices. Today’s consumer is looking for not only convenient food, but above all health-promoting food. The scientific literature describes the possibility of using both mushroom extracts [8] as well as dried mushrooms in ground form for food fortification [9].

In order to use the health-promoting potential of mushrooms, they should be added to staple foods that are consumed by a large part of the general population. This type of food includes bakery products. The available scientific literature describes the possibility of using mushrooms, mainly in a dried and ground form, in the supplementation of breads [10], cakes [11,12], cookies [13], biscuits [14], breadsticks [15] and water extracts in muffins [16]. The addition of mushrooms in various forms or substances isolated from them to food should not exhibit a negative effect on the quality characteristics of the final product. For example, too high a proportion of mushroom powder may have an adverse effect on some baking parameters, which is primarily related to a different protein profile in mushrooms, and it is the gluten present in wheat flour that is responsible for its baking quality [17]. Therefore, it is important to find a compromise between obtaining a food product offering health benefits and, on the other hand, a product acceptable to the consumer in terms of basic technological quality such as colour or texture, which often play a key role in the selection of food products. As the research shows, the addition of dried mushrooms (*P*. *ostreatus*) to various food matrices such as pasta and flatbread may reduce or increase the acceptability of supplemented food, respectively [18,19]. Therefore, when designing new functional food products, both the nutritional and health-promoting value as well as their sensory features should be taken into account.

A simple way to enrich bakery products with mushrooms is to add them in powdered form. Mushroom powders are obtained by grinding previously dried fruiting bodies. Drying mushrooms is one of the most popular methods of their preservation, which is necessary in the case of such perishable raw materials. Mushrooms can be dried by different methods and dried mushrooms are convenient for long-term storage and transportation. The choice of drying method undoubtedly affects the physicochemical characteristics of the mushroom powder. The two most popular drying methods are convection drying, i.e., hot air drying, and freeze-drying. Hot air drying is most often used for mushroom preservation, mainly due to much lower costs compared to freeze-drying [20]. However, by using low temperature dehydration such as in the case of lyophilization, a high-quality product is obtained. Freeze-drying mushrooms is recommended over air drying due to its higher levels of antioxidant compounds [21]. Freeze-drying resulted in better retention compounds having higher total phenolic content, DPPH scavenging activity and reducing power assay in freeze-dried samples of button mushrooms than samples drying at 50 °C [22]. Moreover, the drying methods had significant impact on the colour profile of mushrooms. Maillard reaction products are formed as a result of raw materials containing free amino acids and sugars being exposed to high temperatures, which takes place when drying mushrooms with hot air [23]. It was observed that the mushroom samples dried by cabinet drying (at 50 °C) were slightly darker than the freeze-dried samples showing lesser L* values measured by CIE-L*a*b* colour space [22]. The use of darker mushroom powders can have a large impact on the overall change in colour of the supplemented bread, which is an essential attribute influencing the acceptability of a product by consumer.

The use of dried mushrooms for the supplementation of food products also has benefits in terms of the possibility of increasing the health-promoting value of dried mushrooms. Mushrooms are a suitable raw material for enriching food with ergocalciferol [15]. The results presented by Sławińska et al. [24] show that it is possible to convert ergosterol to vitamin D_2_ in processed mushrooms (hot air dried or lyophilized), which provide an opportunity to obtain new sources of vitamin D with its high content. A higher content of ergocalciferol is obtained in freeze-dried mushrooms than in hot air dried mushrooms, which may be related to the different structure of mushrooms dried by various methods. The lyophilisates are characterized by the presence of a large number of pores. Such a pore structure can facilitate the penetration of UVB radiation and causes a higher level of conversion of ergosterol in the lyophilized mushrooms [24]. Moreover, mushrooms and yeasts are the only non-animal-based food containing vitamin D for vegetarians and vegans and are also a good alternative for people with lactose intolerance and looking for foods rich in vitamin D, especially since most of the enriched foods with the vitamin are dairy products [2].

Studies have been published showing the possibility of using UV-irradiated yeast for baking bread with an increased content of vitamin D [25]. UV-irradiated baker’s yeast (*Saccharomyces cerevisiae*) for the production of fortified yeast-leavened bread and baked goods was approved as a Novel Food Ingredient in the European Union, according to Regulation (EC) No. 258/97 [26]. Recently, the EU approved the request for the production and marketing of a novel food product: UVB-exposed fresh mushrooms [27] and mushroom powder with increased content of vitamin D_2_ [28,29].

According to Płudowski et al. [30], the recommended daily dose (RDA) of vitamin D for healthy groups in the Central European population ranges from 10 µg (for neonates and infants) to 50 µg (for adults and the elderly over 65 years of age). The panel of experts [30] also decided to adopt the Tolerable Upper Intake Level (UL) of vitamin D recommended by the European Food Safety Authority presented in the document “Scientific opinion on the Tolerable Upper Intake Levels of vitamin D for relevant population groups” [31]. UL for individual groups is as follows: 25 µg for healthy neonates and infants (0–6 months), 50 µg for children (1–10 years), 100 µg for adolescence (11–18 years), and adults and seniors with normal weight and 250 µg for overweight seniors and adults. Taking into account the listed RDA and UL levels, it is important to adjust the amount of mushroom powder used with increased vitamin D content to the supplementation of bread. Despite the fact that the breads consumption in Europe has been on the decline in the past few years, bread is still a staple food in many countries [32]. The proposed level of vitamin D_2_ for novel foods is 2.25 µg per 100 g of non-beverage products [27].

The average consumption of bread in Europe is 170 g/day; however, the consumption of this type of products has been decreasing in recent years [32]. Cereals are rich in minerals, but the bioavailability of these minerals is usually low due to the presence of antinutritional factors such as phytate and polyphenoles [33]. Edible mushrooms are a better source of some minerals, e.g., of zinc than plants (according to WHO/FAO standards) [34]. Moreover, bioavailability in vitro of minerals from mushrooms using artificial digestive juices is confirmed by research [35].

In Poland, as with the rest of Europe, the most popular mushrooms, which are available all year round, are button mushrooms (*Agaricus bisporus*). *A*. *bisporus* also is one of the four most popular fungal species in the world [36]. There are several varieties of button mushrooms available for sale that differ in colour, size and maturity of the fruiting bodies: white, cremini, and portobello. In recent years, there has been a growing interest in the brown variety (cremini). In Europe, there is a commonly held belief that cremini mushrooms are more aromatic and tasty, and that they are characterized by a higher nutritional value than traditional white button mushrooms [37]. The general availability of button mushroom varieties throughout the year, their popularity among consumers, cheap raw material purchase costs in relation to other cultivated mushrooms such as oyster mushroom (*Pleurotus ostreatus*) or shiitake (*Lentinula edodes*), make it possible to believe that button mushroom fruiting bodies are a suitable raw material for bread supplementation. Moreover, among the three common species of cultivated edible mushrooms: *A. bisporus*, *P*. *ostreatus* and *L*. *edodes*, *A*. *bisporus* possessed the highest antioxidant activity and total polyphenol content [38]. Additionally, dried button mushrooms were characterized by the highest amount of ergocalciferol after irradiation with UVB radiation, compared to oyster or shiitake mushrooms [24].

In this study, the objective was to substitute 2.5% and 5% of wheat flour with mushroom powder from white and brown (cremini) button mushrooms. The final bread samples were evaluated and compared with the control to determine the effect of mushroom powders on basic technological quality (colour parameters, specific volume and texture), sensory evaluation as well as on health promoting properties of breads such as antioxidant activity (DPPH, FRAP), total polyphenols and vitamin D_2_ content. To obtain the vitamin D_2_-rich mushroom powder for bread supplementation, the lyophilisates were previously exposed to UVB radiation. To the best of our knowledge, we are the first to present the possibility of using UVB-irradiated *Agaricus bisporus* powders obtained from two popular varieties of this mushroom: white and brown.

## 2. Materials and Methods

### 2.1. Mushroom Processing

The mushrooms were processed, as described by Sławińska et al. [24]. Mushrooms (*Agaricus bisporus* (Lange) Sing.) of white and brown (cremini) varieties were obtained from local producers and processed within 12 h. Fresh mushrooms were sliced (3–4 mm) and frozen at −22 °C for 24 h and then freeze-dried for 72 h (Christ Alpha 1-2 LD plus, Martin Christ Gefriertrocknungsanlagen GmbH, Osterode am Harz, Germany). Mushroom lyophilisates were irradiated with a UV lamp (Uvitec LF 206LM, Cambridge, UK) within 12 h from the end of the drying process. For this purpose, about 20 g of dried mushrooms were spread in a single layer on an area of crate (28 × 38 cm) and placed under a UV lamp installed 30 cm above the surface of the mushrooms. The process of irradiation with UVB radiation was carried out for 30 min at a temperature of 20 °C at a wavelength of 312 nm. Subsequently, lyophilized mushrooms were ground to a fine powder using a mill (8000 rpm, 4 min) (Retsch GM200, Retsch GmbH, Haan, Germany). The powder obtained was sieved using 0.25 mm sieve.

### 2.2. Bread Making

The flour used for the preparation of one loaf white bread control was bread wheat flour (400 g), type 650 (average 0.65% ash content, water content 14% wet basis). Part of the flour 2.5% or 5% (*w*/*w*) were replaced by lyophilized and milled mushrooms prepared, as described in Section 2.1. Mushroom processing. Furthermore, for the production of one loaf 7 g of instant yeast, 6 g of salt and 200 mL of water were added. All of these ingredients were mixed using a device Kenwood Chef XL Elite. The dough was kneaded with the hook for 10 min at speed level 3. Shaped dough pieces were placed in moulds with the dimensions 7 cm × 10 cm × 20 cm. The direct, single-phase method was used to bake the bread. Prepared dough was fermented at 30 °C for 50 min. Afterwards, the bread was baked at 240 °C for 8 min, then set aside the door of the oven for 2 min and finally the bread was baked at 190 °C for 30 min. After baking, the breads were cooled down at room temperature and then analysed. 

The samples were designated as: Control—bread without addition of mushroom powder; AbW2.5—bread with 2.5% addition of *Agaricus bisporus* white; AbW5—bread with 5% addition of *Agaricus bisporus* white; AbB2.5—bread with 2.5% addition of *Agaricus bisporus* brown; AbB5—bread with 5% addition of *Agaricus bisporus* brown.

### 2.3. Technological Quality of Bread

#### 2.3.1. Colour Measurement

Colour of crumb and crust of bread enriched with mushroom powder was tested using 3Color K9000 Neo (3Color, Cracow, Poland). The measurements were performed using a D65 light source and a standard colorimetric observer with a 10° field of view. CIE-L*a*b* colour space was used for evaluation of L* (whiteness or darkness), a* (greenness or redness), b* (blueness or yellowness), accordingly [39]. The colour analysis was performed in 15 replicates. The results were expressed as mean value ± standard deviation (SD). The total colour change (ΔE) was the parameter considered for the overall colour difference evaluation between a sample with and without the addition of mushroom powder (control). ΔE was calculated as a colour change index according to the equation [40]:

ΔE = [(L*_sample_ − L*_control_)^2^ + (a*_sample_ − a*_control_)^2^ + (b*_sample_ − b*_control_)^2^]^½^ (1)

The browning index (BI) for crust were calculated on the basic of the following equation [41]:

BI = 100 − L*(2)

#### 2.3.2. Texture Profile Analysis (TPA)

Measurements were performed with a TA-XT2i Texture Analyser (Stable Micro Systems, Godalming, UK). The bread samples (central part of bread, cylindrical, sample size −10 mm in diameter and 15 mm in height) were double compressed to 50% of deformation by a testing set (35 mm diameter) according to the protocol described by Sołowiej et al. [42]. The following operating parameters were used: pre-test speed: 5 mm/s; test speed: 1 mm/s; post-test speed: 5 mm/s; delay between the compressions: 5 s. Bread samples were evaluated for hardness, adhesiveness, springiness and cohesiveness, using Texture Expert software. Five measurements were carried out for each of the three replicates on samples cut from the middle part of the bread and average reported. The results were expressed as mean value ± standard deviation (SD).

#### 2.3.3. Bread Loaf Volume

The volume of loafs was determined by the rapeseed displacement method [43]. Each loaf was put in a container and covered with rapeseeds to fill the container. After that, the loaf was removed, and the volume of rapeseed was recorded. The results were expressed as mean value ± standard deviation (SD).

#### 2.3.4. Specific Loaf Volume

The specific volume (cm^3^/g) was determined as bread volume divided by the weight of bread. The results were expressed as mean value ± standard deviation (SD).

### 2.4. Determination of Vitamin D_2_ in Breads and Mushroom Lyophilisates

#### 2.4.1. Extraction Procedure

The extraction of ergocalciferol was carried out by the method of Ko et al. [44] with minor modifications [24]. The breads were cut into 1 cm thick slices, frozen at −22 °C for 24 h and then lyophilized in a freeze dryer (Christ Alpha 1-2 LD plus, Martin Christ Gefriertrocknungsanlagen GmbH, Osterode am Harz, Germany) for 48 h. Irradiated-mushroom lyophilisates and breads were ground to a fine powder using a mill (Retsch GM200, Retsch GmbH, Haan, Germany) and sieved (0.25 mm). Powdered mushrooms or breads in an amount of 1 g were placed in a 250 mL flask, then cholecalciferol (vitamin D_3_) (Sigma Aldrich, St. Louis, MO, USA) was added as an internal standard. Subsequently, reagents were added to the flask in the following order: 1 g of L-ascorbic acid (Chempur, Piekary Śląskie, Poland) as an antioxidant, 50 mL of 96% ethanol (POCH, Gliwice, Poland) and finally 25 mL of 50% potassium hydroxide (POCH, Gliwice, Poland). The saponification process was carried out for 0.5 h at 80 °C. After the mixture was saponified and cooled, it was transferred to a separating funnel. Subsequently, the mixture was extracted twice by adding 10 mL of deionized water and 30 mL of n-hexane (Sigma Aldrich, St. Louis, MO, USA). In order to neutralize the collected hexane layer, it was washed three times with deionized water. Next, the organic layer with an added 2 mL of BHT solution (1 mg/mL in hexane) (Merck, Darmstadt, Germany) and 12 mL of 99.8% ethanol (POCH, Gliwice, Poland) was transferred into a 100 mL flask, rotary evaporated to dryness at 40 °C and immediately redissolved in 3 mL of solvents A and B mixed in ratio 2:1, where A is mixture of methanol/acetonitrile (3:1 ratio) and B is isopropyl alcohol. Subsequently, the obtained samples were centrifuged for 10 min at 16,000× *g* (MPW-350R, Warsaw, Poland) at 20 °C. The supernatants were subjected to UHPLC/MS/MS analysis.

#### 2.4.2. UHPLC/MS/MS Analysis

Ergocalciferol was chromatographically analysed using an Agilent 1290 Infinity series ultra-high performance liquid chromatograph (Agilent Technologies, Santa Clara, CA, USA) coupled to a 6460 triple quadrupole spectrometer (Agilent Technologies, Santa Clara, CA, USA) according to the protocol described by Sławińska et al. [24]. The UHPLC system consisted of a binary pump, an autosampler, a thermostat and a DAD detector. The spectrometer was equipped with a Jet Stream technology ion source. Chromatographic separation was performed on Zorbax Plus C18 analytical column, 2.1 × 100 mm, 1.8 µm particle size (Agilent Technologies, Santa Clara, CA, USA) at 30 °C. LC-MS grade methanol was used as a mobile phase, flow rate 0.3 mL/min, analysis time 5 min. The injection volume was 1 µL. The UV-signal was registered at 264 nm. The electrospray source was operated in positive ion mode; the fragmentor was set at 80 V, gas temperature 325 °C, gas flow 8 L/min, nebulizer gas 35 psi, sheath gas temperature 250 °C, sheath gas flow 11 L/min, capillary voltage 3500 V, nozzle voltage 500 V. Ion acquisition was carried out in the multiple reaction monitoring (MRM) mode. For ergocalciferol transitions of ions of *m*/*z* 397.3 to ions of *m*/*z* 271.3 (collision energy, CE 10 eV), 124.9 (CE 10 eV) and 69.1 (CE 30 eV) were monitored. For cholecalciferol transitions of ions of *m*/*z* 385.0 to 259.1 (CE 10 eV) and 159.1 (CE 30 eV) were monitored. Retention times, based on MS-signal, were 3.2, 3.3 for ergocalciferol, cholecalciferol, respectively. Agilent Mass Hunter software version B.06 was used for data acquisition, instrument control and data analysis.

### 2.5. Determination of Total Phenolic Content (TPC) and Antioxidant Properties

#### 2.5.1. Extraction Procedure

Dried mushrooms fruiting bodies and bread samples were powdered in a mill (Retsch GM200, Retsch GmbH, Haan, Germany) and sieved through the appropriate 0.25 mm screen before the extraction procedure. Ethanol (80% *v*/*v*) was used as the solvent. The samples (1 g) were extracted with 30 mL of a solvent in a shaker at 80 °C, 180 rpm for 1 h according to the protocol described by Radzki et al. [45]. The extracts were separated by centrifugation at 4200× *g* for 20 min (MPW-350R, Warsaw, Poland).

#### 2.5.2. Determination of Total Phenolic Content (TPC)

The concentration of phenolic compounds in the extracts were measured according to the method of Singleton and Rossi [46] with some modifications [46]. The samples (0.2 mL) were mixed with 0.8 mL Folin and Ciocalteu’s phenol reagent (10 times diluted). After 3 min, 1.25 mL of 7% Na_2_CO_3_ was added to the mixture. The reaction was kept in the dark for 30 min and an absorbance was read at 725 nm (Helios Gamma, Thermo Fisher Scientific, Waltham, MA, USA). The calibration curve was constructed with different concentrations of gallic acid as a standard and total phenolics content was expressed as a gallic acid equivalent (GAE) per 1 g of mushroom or bread dry weight (dw). The results were expressed as mean value ± standard deviation (SD).

#### 2.5.3. Antioxidant Activity

##### DPPH

The scavenging ability of 1,1-diphenyl-2-picrylhydrazyl (DPPH) radicals was conducted using the method of Choi et al. [47]. The extracts (0.2 mL) were mixed with 0.8 mL of DPPH ethanolic solution (0.2 mM) and the mixture was shaken vigorously before it was left to stand for 15 min in the dark. The absorbance was measured at 520 nm against the blank sample. The calibration curve was performed with different concentrations of Trolox as a standard and the antioxidant potential was expressed as µmol Trolox equivalent (TE) per 1 g of mushroom or bread dry weight. The results were expressed as the mean value ± standard deviation (SD).

##### FRAP

Antioxidant capacity was also determined by the ferric-reducing antioxidant assay (FRAP) described by Thetsrimuang et al. [48]. FRAP reagent was prepared by mixing 300 mM acetate buffer (pH 3.6) with 10 mM 2,4,6-tripyridyl-striazine (TPTZ) solution in 40 mM HCl and 20 mM FeCl_3_·6H_2_O (10:1:1 ratio). Assay solutions were prepared by mixing 1.9 mL of FRAP reagent with 0.1 mL of mushroom extract. The mixtures were then incubated at 37 °C in the dark for 15 min. The amount of ferrous tripyridyltriazine complex was measured by reading the absorbance at 593 nm. The calibration curve was constructed with different concentrations of Trolox as a standard and the results were reported as µmol Trolox equivalent (TE) per 1 g of mushroom or bread dry weight. The results were expressed as the mean value ± standard deviation (SD).

### 2.6. Dry Weight Determination

The results were expressed on a dry weight basis (dw), due to the different moisture content of the samples. The samples were dried in a laboratory dryer at 105 °C until a constant weight was obtained [45].

### 2.7. Sensory Evaluation

Breads with different percentages of mushroom powder was submitted to a panel of 30 people (aged between 21 and 50 years old). Untrained consumers were recruited among students and staff at Life Sciences University in Lublin. The sensory evaluation was carried out with bread samples 6 h after baking. Coded samples of breads (5 × 5 × 1 cm) with a random 3-digit number were given to the evaluation panel on white plates. Water was used to rinse the mouth before and after each sample test. A nine-point hedonic scale was used to determine the degree of overall liking or disliking of different types of bread, where 1 = dislike extremely, 5 = neither like nor dislike, 9 = like extremely). Bread samples were evaluated for acceptance of attributes: crumb colour, aroma, texture, taste and overall acceptability [49].

### 2.8. Statistical Analysis

Statistical analysis was performed using the STATISTICA 13.3 software (StatSoft, Cracow, Poland), applying the Tukey test in the analysis of variance (one-way ANOVA) to estimate the significance of the differences between the mean values at *p* < 0.05. Pearson’s correlation coefficients were calculated to evaluate the relationships between analysed parameters (between the amount of used mushroom powder and the colour parameters of crust and crumb (i), the total phenolics content (ii), the antioxidant properties (DPPH (iii), FRAP (iv)), the content of vitamin D (v), and between the total phenolics content and the antioxidant properties (DPPH (i), FRAP (ii) of the bread samples) and considered significant at the level of *p* < 0.05.

## 3. Results and Discussion

### 3.1. Colour Measurements

Table 1 shows the L*, a*, b*, BI and ∆E for the breads and L*, a*, b* for mushroom powders. The colour parameters of the white and brown button mushroom powders varieties differed significantly. The L*, a*, and b* values in mushroom lyophilisates were 86.19, 1.66, and 15.29 in the white mushrooms powder and 75.59, 4.95, and 20.28 in the brown mushroom powders, respectively.

The lightness (L*) of fresh white button mushrooms reaches values in the range of 80–85 [50] or above 90 [51]. According to Jabłońska-Ryś et al. [52], the caps colour parameters of fresh white and brown button mushrooms were significantly different. White mushrooms were characterized by higher L* values (89.02) and lower a* (0.73) and b* (12.8) values. The L*, a* and b* colour parameters for the brown variety were 58.51, 9.30, and 16.21, respectively.

Lower values of the L* parameter and higher values of the a* parameter of the powders obtained from the brown button mushroom may result not only from the brown caps colour but also may result from the greater susceptibility of the flesh to enzymatic browning. The study by Bernaś [53] showed that the brown variety was less resistant than the white variety to enzymatic browning, due to a higher activity of monophenolase and a minor degree of diphenolase. The browning of fruiting bodies takes place as a result of enzymatic degradation of phenols to quinones, which condense to dark melanins [51]. Additionally, UV radiation can lead to a darkening of mushroom caps [54].

The crusts of the bread supplemented with brown mushroom had a darker colour. The results showed that the L* value of the crust decreased significantly with an increase in *A*. *bisporus* powder, with one exception (AbW2.5). A stronger negative correlation between the amount of mushroom powder and the L* parameter was found for breads with brown button mushroom lyophilisates (r = −0.853; *p* < 0.05; *n* = 30) than for white mushrooms (r = −0.620; *p* < 0.05; *n* = 30). In the case of the crust, the L* parameter value was significantly lower for the samples with the addition of freeze-dried mushrooms compared to the control, except for the samples marked with the AbW2.5 symbol, for which the L* and a* parameter values did not differ statistically. A statistically significant correlation (*p* < 0.05; *n* = 30) was found between the amount of mushroom powder added and the b* parameter for crusts, both for breads with white mushroom powder (r = 0.750) and for bread enriched with brown mushroom powder (r = −0.606). The crust of the bread supplemented with the brown button mushroom powders was darker than the crust of the bread with the addition of white button mushroom lyophilisates. Bread crusts with the addition of cremini mushroom powders, in addition to lower values of L* and a* parameters, were also characterized by higher values of BI parameter.

Colour measurements of crumbs showed lower L* values in breads with higher white or brown mushroom powder addition (r = −0.90; *p* < 0.05; *n* = 30 and r = −0.97; *p* < 0.05; *n* = 30, respectively). For white bread (control) the L* value was the highest and reached 75.59. Among the crumbs of the supplemented breads, the value of L* decreased with the increasing addition of mushroom powder. The redness of the samples increased as the addition of mushroom lyophilisate increased (r = 0.98; *p* < 0.05; *n* = 30). Greenness–redness balance for the crumbs of the breads varied form 1.02 for control samples to 5.58 for breads with 5% brown button mushrooms powder (AbB5) (r = 0.12; *p* < 0.05; *n* = 30 and r = 0.28; *p* < 0.05; *n* = 30 for white and brown mushroom powder, respectively). With regard to the parameter b*, the addition of 5% mushrooms powder to breads samples caused an increase in yellowness. On the other hand, the bread with a 2.5% mushroom powder addition had lower values of parameter b* than the control samples.

The highest total colour difference ΔE was recorded for the samples with a 5% addition of brown mushrooms (AbB5) for the crumb and crust (19.51 and 14.88, respectively).

Similar results were obtained by other authors; bread enriched with mushroom button powder was characterized by lower L* values for the crumb and crust, higher a* and b* parameters for the crumb, and lower a* and b* parameters for the crust of the breads [55]. Ulziijargal et al. [56] report that a 5% addition of the lyophilisates of five mushroom species caused a decrease in the L* parameter and an increase in a* and b* parameters. Gaglio et al. [57] studied the effect of the addition of *Pleurotus eryngii* lyophilizateto bread and showed that supplementation in the amount of 5% or 10% causes a decrease in L* parameters and an increase in the a* and b* parameters for the crumb and crust and a decrease in the b* parameter for the crust.

The change in colour of bread with mushrooms may be related, firstly, to the pigments of the mycelium or oxidation of the phenolic compounds of the mycelium during baking, and secondly, to more intense Maillard reactions during thermal processing, which may be the result of a larger amount of soluble sugars and free amino acids in the mycelium than wheat flour [56]. According to Djordjević et al. [58], the crust colour associated is mainly the result of Maillard and caramelisation reactions at high temperatures, while the crumb colour depends primarily on the colour of the ingredients used.

### 3.2. Texture Profile Analysis (TPA)

The effect of different types and concentrations of lyophilized mushrooms on the textural properties of bread are presented in Table 2. In general, the hardness of bread increased significantly (*p* < 0.05) with an increase in mushroom (white or brown button mushrooms) concentrations, whereas cohesiveness and springiness of tested samples decreased. The control samples were characterized by the lowest hardness (1710 g) and the highest springiness and cohesiveness (0.86 and 0.56, respectively). The 5% addition of all types of lyophilized mushrooms led to the highest hardness of tested bread and the lowest cohesiveness and springiness. However, only the results for the springiness parameter of bread with the addition of white mushroom differ statistically from those for control bread. Nevertheless, the values of adhesiveness of all tested breads were negligible.

As the research of other authors shows, replacing a part of wheat flour with mushroom powder results in an increase in the hardness of the crumb [10,55,59], and decrease in cohesiveness [55] and springiness [10,55,59]. In our studies, the upward trends in the hardness and decrease in the cohesiveness and springiness of the evaluated breads could be attributed to a decrease in the specific volume (Table 2). The increase in hardness is probably related to the different chemical composition of the mushroom powders compared to wheat flour, which contains gluten [60] and the addition of dried mushrooms may interfere with the optimal gluten matrix formation during fermentation and baking [61]. Research published by the authors of Ulziijargal et al. from 2013 [56] shows that the addition of 5% dried mushrooms of different species may result in different results on some texture profile analysis parameters of fresh bread, e.g., supplementation with *Agaricus blazei* or *Hericium erinaceus* causes a decrease in springiness, while the addition of *Antrodia camphorata* and *Phellinus linteus* results in an increase in springiness compared to bread made with 100% wheat flour. Additionally, breads with *P*. *linteus* mycelium showed less hardness than control samples, while the addition of lyophilisates of other species resulted in an increase in this parameter.

### 3.3. Specific Loaf Volume

Mean values related to specific loaf volume are shown in Table 2. According to the results, specific volume varies from 3.42 to 2.67 cm^3^/g. The highest specific values were noted for bread made with 100% wheat flour. The mean values for a specific loaf of bread prepared from a different amount of mushroom powder were lower compared to mean value for that parameter of control bread. However, statistical analysis showed that the addition of 2.5% of white button mushroom lyophilisate had no significant effect (*p* < 0.05) on the reduction of the specific loaf value in comparison with the control samples. Additionally, breads supplemented with brown button mushrooms powder were characterized by a lower specific value than bread with the addition of white button mushrooms.

A reduction in discussed parameter of breads enriched with mushroom powder was also described in several publication. The findings of Ulziijargal et al. [56] and Gaglio et al. [57] illustrated that the specific volume of bread enriched with mushroom lyophilisate was much lower than bread control and resulted in a smaller size loaf. Lower specific volume values were noted when a part of wheat flour was replaced in an amount from 2% to 20% with various dried mushrooms, e.g., button mushrooms [55,59], oyster mushroom [17], or shiitake [59]. On the other hand, the same studies Lu et al. [59] show that the addition of porcini mushrooms in smaller amounts (5%) increases the specific volume; however, the addition of 15% significantly decreases the value.

As written above, supplemented breads with button mushrooms showed a lower specific volume and denser breadcrumbs, which could explain the higher crumb hardness. A similar negative correlation between hardness and specific volume was noted in other studies [55,61,62]. The decrease in loaf volume of the bread may be attributed to the reduction in the wheat structure-forming proteins. The protein quantity and smaller amount of gluten in mixed flours, the presence of enzymes in mushrooms that can hydrolyse proteins and starch, might have a significant effect on bread volume and baking quality for different composite flours [17,63]. Moreover, the introduction of nutrients into the yeast dough together with wheat flour substitutes has an impact on the growth of yeast and the production of CO_2_, thus influencing loaf volume [64].

### 3.4. Total Phenolic Content and Antioxidant Properties

Table 3 shows the results of total polyphenols content and antioxidant properties of mushroom powders and breads. Mushrooms powder from brown button mushrooms were characterized by slightly higher level of total phenolic content (11.31 mg GAE/g dw) compared with white button mushroom powder (10.44 mg GAE/g dw); however, the statistical analysis did not show any significant differences. The antioxidant activity measured by the DPPH method was recorded at a similar level for the white and brown mushroom powders (15.15 and 15.06 µmol TE/g dw, respectively). Results showed that white button mushroom powder had a statistically significant higher reducing activity (18.06 µmol TE/g dw) compared to the cremini mushroom powder (16.84 µmol TE/g dw).

The studies have shown that the breads with the addition of mushroom powders characterized by a higher antioxidant property and a higher total polyphenol content (TPC) compared to bread control (Table 3). The total polyphenol content for all tested samples ranged from 0.76 mg GAE/g dw (control bread) to 1.25 mg GAE/g dw for the samples with a 5% addition of mushroom lyophilisate. The amount of mushroom powder added had a statistically significant impact on the increase in TPC content in supplemented breads, with a higher total polyphenol content for the samples with brown button mushroom powder compared to the breads with 2.5% of white button mushroom lyophilisates (1.11 and 0.98 mg GAE/g dw, respectively).

It was noted that the antioxidant activities were higher for breads supplemented with mushrooms powder. The control bread had the lowest antioxidant properties measured by DPPH and FRAP methods (0.88 µmol TE/g dw and 0.97 µmol TE/g dw, respectively). Regardless of the amount of white and brown mushroom lyophilisate used (2.5% and 5%), there were no significant statistical differences in the level of antioxidant properties measured with the DPPH method. These values ranged from 0.94 to 1.20 µmol TE/g dw. Significant statistical differences between the control sample (0.88 µmol TE/g dw) and the enriched samples were noted with the addition of the lyophilisate at the level of 5% (1.18 and 1.20 µmol TE/g dw for white and brown mushrooms, respectively).

It was noted that antioxidant activity measured by the FRAP method increased significantly with the increasing substitution level of *A*. *bisporus* powder. The control bread had the lowest antioxidant properties measured by the FRAP method (0.97 µmol TE/g dw), and the addition of mushrooms powder, regardless of whether it was a lyophilisate of white or brown button mushrooms, in the amount of 2.5% or 5% significantly increased the antioxidant activity (from 1.22 to 1.59 µmol TE/g dw).

There is a statistically significant correlation (*p* < 0.05; *n* =18) between the total content of polyphenols of the tested samples and the antioxidant properties measured by DPPH and FRAP methods. The strongest correlation was between TPC, and antioxidant properties measured with the FRAP method (r = 0.891 and r = 0.766, respectively, for bread enriched with white and brown button mushroom lyophilisate), then between TPC and antioxidant properties measured by DPPH method for samples enriched with brown mushroom lyophilisate (r = 0.72). There was also a statistically significant correlation between the total content of polyphenols and the antioxidant properties measured with the DPPH radical for bread with the addition of white button mushrooms (r = 0.589; *p* < 0.05; *n* =18).

The available literature provides many reports of the phenolic compound content and antioxidant activity of fresh button mushrooms. Jabłońska-Ryś at al. [51] report a lower content of polyphenols in white button mushroom (4.87 mg of GAE/g dw) compared to the results presented in this study. On the other hand, similar contents of phenolic compounds for different button mushroom varieties (from 8 to 12.3 mg/g dw) were reported by other authors; however, higher amounts of TPC were recorded for the brown variety [65,66]. Additionally, the antioxidant activities were higher for the brown variety measured by ABTS, DPPH, FRAP, ORAC, NORAC and SORAC methods [65,66].

According to Irakli et al. [67], in wheat bread, antioxidative activity was low, with phenolic and flavonoid being the main group of compounds responsible for these properties. Czaja et al. [68] report that the amount of polyphenols in wheat bread is low (0.3 mg GAE/g dw), which results in low values of antioxidant activity. As previous studies have shown, mushrooms are characterized by a high total polyphenol content and antioxidant properties [4,7,69,70]. Our research shows that replacing even a small amount of wheat flour with mushroom powder significantly increases the total polyphenol content and the antioxidant activity of bread. Similar dependencies were observed by other authors. In the report by Lu et al. [71], the authors studied the effect of replacing wheat flour with mushroom powder (*Agaricus bisporus*, *Lentinula edodes* and *Boletus edulis*) in amounts of 5%, 10% and 15% on the total phenolic contents and antioxidant capacities. Control bread had the lowest total phenolic, DPPH radical-scavenging and ORAC levels. The mushroom powder used as a substitute for wheat flour increased the total phenolic content and antioxidant properties of supplemented breads. Moreover, there was a positive correlation between TPC and both DPPH and ORAC (above 0.96). Zhang et al. [55] report that the bread with the addition of 8% *A*. *bisporus* powder had the highest polyphenol content (64.16 mg GAE/100 g) and more than a twofold increase in TPC compared to the control sample (28.34 mg GAE/100 g). The authors noted that antioxidant activity measurement by DPPH and FRAP methods increased significantly with the increasing substitution level of mushroom powder (from 0% to 8%). Additionally, the addition of polysaccharides from *Auricularia auricula* to bread increases the antioxidant properties of the supplemented breads [72]. These molecules have been proved to exert antioxidant activities. It well known that polyphenols tend to bind with polysaccharide and contribute to their antioxidant activity [38,69].

Heat treatment of food can release insoluble bound phenolic compounds, on the other hand, high temperature can accelerate the destruction of phenols [73]. In addition, under the influence of high temperature (e.g., baking), compounds are formed that affect the antioxidant activity of bread, such as the products of the Maillard reaction [74]. As written by Świeca et al. [74], lower than expected results of phenolic contents and antioxidant activity of breads supplemented with a polyphenol-rich flour substitute may be the result of blocking of reactive groups of polyphenols by bread components. UV radiation may lead to changes in the total polyphenol content and in antioxidant properties of mushrooms [54,75]. Abiotic stress caused by a harmful factor (UV radiation) causes the activation of biochemical pathways and the synthesis of secondary metabolites, e.g., polyphenols and terpenes [76].

### 3.5. Vitamin D_2_

The content of vitamin D_2_ in the tested samples is shown in Table 3. After irradiation with UVB radiation of mushroom lyophilisates for 30 min, statistically higher amounts of ergocalciferol were found in the lyophilisate of brown button mushroom (276.5 μg/g dw) compared to the lyophilisate of white button mushroom (193.2 μg/g dw), which was reflected in the amount of this compound in supplemented breads. As expected, no ergocalciferol was found in the control bread samples. Breads with 2.5% and 5% addition of brown button mushroom lyophilisate were characterized by a much higher content of vitamin D_2_ (5.93 and 11.99 μg/g dw, respectively) compared to breads enriched with white button mushroom lyophilisate in the same amount (3.75 and 7.76 μg/g dw, respectively).

Irradiating dried mushrooms with UVB radiation is an easy and efficient way to increase their vitamin D content [24]. As previous studies show, the retention of ergocalciferol in mushrooms depends on the temperature of the thermal treatment and on the type of treatment: frying, baking or cooking [77,78]. According to Ložnjak and Jakobsen [77], the retention of vitamin D_2_ was significantly lower than 100% with retention rates ranging from 62% to 88% (for boiling and pan-fried mushrooms at low temperatures, respectively). Moreover, the use of higher temperatures had a negative effect on vitamin D retention. In the earlier studies by these authors, the retention of vitamin D (derived from vitamin D_2_-enriched dry yeast) in rye bread was determined to be lower (73%) than in white bread (85–89%) [78]. Gaglio et al. [57] report that the increase in the amount of *Pleurot eryngii* lyophilisate in wheat bread resulted in a higher content of B vitamins and vitamin D (from 0.09 mg/100 g in bread with 5% to 0.11 mg/100 g in bread with 10% of mushrooms powder). As expected, the breads processed from 100% wheat flour did not contain the vitamins: biotin, B_12_ and D.

### 3.6. Sensory Evaluation

**Figure 1:**
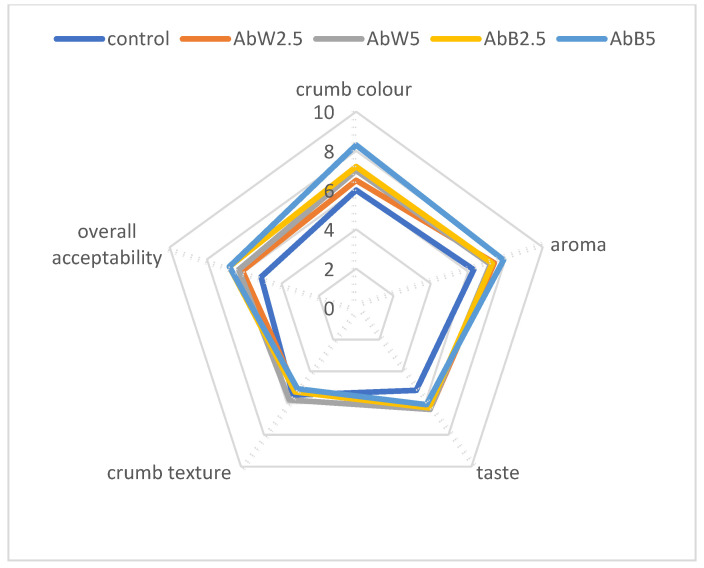
Sensory evaluation of white bread and mushroom powder supplemented bread. Control—bread without addition of mushroom powder; AbW2.5—bread with 2.5% addition of *Agaricus bisporus* white; AbW5—bread with 5% addition of *Agaricus bisporus* white; AbB2.5—bread with 2.5% addition of *Agaricus bisporus* brown; AbB5—bread with 5% addition of *Agaricus bisporus* brown.

Mushroom powder addition to foods elicits sensory attributes that could potentially affect acceptability of product. Figure 1 shows the sensorial attributes of investigated breads evaluated by panellist. When analysing the results, it can be concluded that the addition of up to 5% white or brown mushroom lyophilisate has a positive effect on most analysed attributes. On the nine-point hedonic scale, overall acceptability was in the descending order of: AbB2.5% and AbB5% (6.8) > AbW5% (6.3) > AbW2.5% (6.1) > C (5.1) samples. The control bread also had the lowest scores in overall acceptability (5.1), crumb colour (6.0), aroma (6.3) and taste (5.2) attributes. Attributes such as colour and aroma of bread with the addition of 5% brown mushroom were evaluated the highest (8.3 and 7.9, respectively). The taste of the fortified bread was assessed in the range from 6.1 (AbB5%) to 6.4 (AbW5%). In turn, the texture of all bread variants had scores in the range from 5.1 (AbB5%) to 5.8 (AbW5%). The texture of the 100% wheat bread was rated 5.5. A mushroom’s taste and aroma are dependent on volatile and non-volatile compounds like 5′-nucleotides, free amino acids, organic acids, and soluble carbohydrates [79]. As Bernaś writes in the article from 2017 [37], fresh mushrooms of both varieties differed in the levels of free amino acids, and it should be specified that a higher quantity (by 43–44%) was found in cremini (brown variety) than in button (white variety) mushrooms. Moreover, the level on the EUC index (the equivalent of monosodium glutamate MSG) was higher in cremini mushrooms (by 44%), mainly due to the higher levels of free amino acids, which was also confirmed in the sensory evaluation of taste. This may explain the better scores in our research for the flavour of bread enriched with the freeze-dried cremini mushroom.

This study has not confirmed previous research on impact on sensory evaluation of bread enriched in *Agaricus bisporus* mushrooms powder [55]. Authors reported that the addition of mushroom powder had a negative impact on the acceptability of breads. Samples with a lower mushroom content (2%) showed higher overall acceptability values due to the unique flavour and colour similar to 100% wheat bread, while the addition of mushrooms above 4% drastically reduced the scores. On the other hand, our research included a lower percentage of mushrooms (up to 5%). We know from our previous studies that replacing wheat flour with other substitutes in higher amounts in the production of bakery products may have a negative impact on the sensory evaluation [61]. In this study, supplemented breads with a lower L* parameter and a higher a* parameter received higher scores. The browner colour might attract consumers attention to mushroom-supplemented bread [56]. Gaglio et al. [57] reports that judges rated the colour of crust and crumb breads supplemented with 5% and 10% freeze-dried *Pleurotus eryngii* higher. The judges also provide a score for overall assessment: 3.25, 3.55 and 3.56 for breads with the addition of 0%, 5% and 10% of mushroom powder, respectively. The results presented in the next study show that the addition of *P*. *ostreatus* powder to flatbread increases the acceptability of this product compared to the control samples without the addition of mushrooms [19]. The next two studies indicate that incorporating 5% mushroom powder into the bread formula lowers the bread’s acceptability [56,80]. Ulziijargal et al. [56] suggest that the mycelium-supplemented bread sensory evaluation results could have been higher if the bread was labelled as a new functional product.

## 4. Conclusions

Replacing a small amount of wheat flour in the amount of up to 5% with dried mushrooms significantly increased the content of bioactive compounds (total polyphenols and vitamin D_2_) and the antioxidant properties of bread. Based on the conducted research, it can be concluded that the lyophilisates of white and brown mushrooms can be a valuable raw material for the fortification of bread, which is a good matrix and carrier of substances with documented biological activities. Particularly noteworthy are brown mushroom lyophilisates, with a significantly higher amount of vitamin D_2_, which was reflected in the amount of this compound in the fortified bread. However, further research should be carried out for the usage of mushroom powders with a lower vitamin D_2_ content in order not to exceed the level proposed for novel foods.

According to all presented results, the formulated breads are acceptable for the consumers, regardless of the increased hardness recorded in TPA analysis. However, further research should be conducted to examine the staling process influence on this kind of breads.

## Figures and Tables

**Table 1:** Colour parameters of mushroom powders and mushroom powder-supplemented breads.

Parameters	Mushroom Powder				
	*A. bisporus* white powder			*A. bisporus* brown powder	
L*	86.19 ± 0.27 ^b^			75.59 ± 0.36 ^a^	
a*	1.66 ± 0.07 ^a^			4.95 ± 0.10 ^b^	
b*	15.29 ± 0.25 ^a^			20.28 ± 0.30 ^b^	
	**Bread samples**				
	Control	AbW2.5	AbW5	AbB2.5	AbB5
Crust colour					
L*	59.85 ± 2.41 ^c^	59.03 ± 1.32 ^c^	54.0 ± 1.11 ^b^	55.62 ± 1.86 ^b^	49.34 ± 1.97 ^a^
a*	12.45 ± 1.08 ^b^	12.38 ± 0.72 ^b^	13.94 ± 0.56 ^c^	9.91 ± 0.55 ^a^	9.74 ± 0.66 ^a^
b*	32.07 ± 1.07 ^c^	21.61 ± 1.14 ^a^	31.33 ± 0.80 ^c^	23.90 ± 0.76 ^b^	21.89 ± 1.96 ^a^
∆E	-	10.49	6.08	9.54	14.88
BI	40.15	40.97	46.00	44.38	50.66
Crumb colour					
L*	75.59 ± 1.17 ^e^	72.54 ± 1.72 ^d^	61.20 ± 0.96 ^b^	64.15 ± 1.27 ^c^	56.75 ± 1.51 ^a^
a*	1.02 ± 0.17 ^a^	2.38 ± 0.22 ^b^	3.84 ± 0.16 ^d^	2.77 ± 0.09 ^c^	5.58 ± 0.34 ^e^
b*	21.65 ± 0.57 ^c^	16.22 ± 1.09 ^a^	22.99 ± 0.36 ^d^	17.83 ± 0.27 ^b^	23.84 ± 1.21 ^d^
∆E	-	6.37	14.72	12.19	19.51

L*—lightness. a* (+) redness. (-) greenness. b* (+) yellowness. (-) blueness values are means of 15 replications ± SD standard deviations; ^a–e^—the same letters in row mean that there are no significant differences between mean values at *p* < 0.05 (HSD Tukey’s test); ∆E—total colour change; BI—browning index; Control—bread without addition of mushroom powder; AbW2.5—bread with 2.5% addition of *Agaricus bisporus* white; AbW5—bread with 5% addition of *Agaricus bisporus* white; AbB2.5—bread with 2.5% addition of *Agaricus bisporus* brown; AbB5—bread with 5% addition of *Agaricus bisporus* brown.

**Table 2:** Specific volume and texture profile analysis of breads prepared by partial substitution of wheat flour with mushroom powder.

Parameters	Bread Sample				
	Control	AbW2.5	AbW5	AbB2.5	AbB5
Hardness (g)	1710 ± 169 ^a^	3939 ± 225 ^b^	5582 ± 80 ^c^	3529 ± 99 ^b^	5438 ± 153 ^c^
Adhesiveness (g/s)	−1.45 ± 0.37 ^bc^	−0.91 ± 0.06 ^cd^	−1.88 ± 0.09 ^b^	−0.60 ± 0.05 ^d^	−6.17 ± 0.28 ^a^
Springiness	0.860 ± 0.022 ^c^	0.757 ± 0.035 ^b^	0.517 ± 0.061 ^a^	0.823 ± 0.012 ^bc^	0.789 ± 0.029 ^bc^
Cohesiveness	0.560 ± 0.008 ^c^	0.477 ± 0.024 ^ab^	0.436 ± 0.040 ^a^	0.492 ± 0.023 ^b^	0.425 ± 0.019 ^a^
Specific volume (cm^3^/g)	3.42 ± 0.05 ^c^	3.30 ± 0.05 ^bc^	3.18 ± 0.05 ^b^	2.82 ± 0.10 ^a^	2.67 ± 0.16 ^a^

^a–d^—the same letters in row mean that there are no significant differences between mean values at *p* < 0.05 (HSD Tukey’s test); results are expressed as mean ± SD; Control—bread without addition of mushroom powder; AbW2.5—bread with 2.5% addition of *Agaricus bisporus* white; AbW5—bread with 5% addition of *Agaricus bisporus* white; AbB2.5—bread with 2.5% addition of *Agaricus bisporus* brown; AbB5—bread with 5% addition of *Agaricus bisporus* brown.

**Table 3:** Vitamin D_2_, total polyphenol content (TPC) and antioxidant activity of mushroom powders and mushroom powder-supplemented breads.

Sample	Vitamin D_2_ μg/g dw	TPC mg GAE/g dw	DPPH μmol TE/g dw	FRAP μmol TE/g dw
*A. bisporus* white powder	193.20 ± 4.94 ^A^	10.44 ± 0.28 ^A^	15.15 ± 0.19 ^A^	18.06 ± 0.12 ^B^
*A. bisporus* brown powder	276.50 ± 6.89 ^B^	11.31 ± 0.34 ^A^	15.06 ± 0.78 ^A^	16.84 ± 0.17 ^A^
Control	n. d.	0.76 ± 0.07 ^a^	0.88 ± 0.04 ^a^	0.97 ± 0.06 ^a^
AbW2.5	3.75 ± 0.22 ^a^	0.98 ± 0.02 ^b^	0.94 ± 0.14 ^ab^	1.31 ± 0.04 ^b^
AbW5	7.76 ± 0.07 ^c^	1.25 ± 0.04 ^d^	1.18 ± 0.07 ^b^	1.57 ± 0.03 ^c^
AbB2.5	5.93 ± 0.20 ^b^	1.11 ± 0.03 ^c^	0.97 ± 0.14 ^ab^	1.22 ± 0.04 ^b^
AbB5	11.99 ± 0.61 ^d^	1.25 ± 0.06 ^d^	1.20 ± 0.09 ^b^	1.59 ± 0.08 ^c^

^A,B^ or ^a–d^—the same letters in column mean that there are no significant differences between mean values at *p* < 0.05 (HSD Tukey’s test); results are expressed as mean ± SD; n. d.—not detected; Control —bread without addition of mushroom powder; AbW2.5—bread with 2.5% addition of *Agaricus bisporus* white; AbW5—bread with 5% addition of *Agaricus bisporus* white; AbB2.5—bread with 2.5% addition of *Agaricus bisporus* brown; AbB5—bread with 5% addition of *Agaricus bisporus* brown.

## Data Availability

The data used to support the findings of this study can be made available by the corresponding author upon request.
